# From ‘Lack of Insight’ to Being a Knower: A Personal Turning Point in Psychiatric Inpatient Care

**DOI:** 10.1111/jpm.70175

**Published:** 2026-07-09

**Authors:** Jennie Moberg

**Affiliations:** ^1^ Department of Social Work Stockholm University Stockholm Sweden

## Abstract

**Background:**

At times, the psychiatric inpatient system pathologizes patient perspectives as ‘lack of insight’, framing survival strategies as DSM symptoms. In these moments, power imbalances emerge that risk silencing patients' experiential knowledge.

**Purpose:**

This autoethnography reflects on my journey from invalidated patient to ‘knower’ to underscore the need for curiosity‐driven, person‐centred practices that validate lived wisdom in mental health recovery.

**Methods:**

A first‐person autoethnographic narrative draws on lived experience of inpatient care, including periods of compulsory treatment during severe anorexia and post‐ECT amnesia, combined with retrospective reflection and critical engagement with psychiatric literature and power dynamics.

**Findings:**

When patients' voices are ignored, recovery stalls, risking internalized illness identities. Reclaiming narrative through writing enables ‘double recovery’—from suffering and systemic marginalization. This fosters equality through questions like ‘How do you understand your experience?’—a stark contrast to DSM's symptom‐focused framing.

**Relevance to Mental Health Nursing:**

Drawing on my experience of inpatient care, mental health nursing can help shift psychiatric practice towards a more collaborative approach that actively incorporates patients' own explanations into assessment, documentation and care planning. This can begin by asking how patients understand what is happening, recording their own explanations alongside clinical observations and co‐designing goals that align with patients' priorities. In doing so, staff can better recognize survival strategies as meaningful responses to distress and strengthen trust through more person‐centred practice.

## Summary

**What is known about the subject:**

- Patients in psychiatric inpatient care are often labelled with ‘lack of insight’ when their views differ from clinical perspectives.
- Biomedical approaches prioritize diagnostic categories like DSM, overlooking patients' ways of enduring and embodied knowledge.

**What the paper adds to existing knowledge:**

- It offers an autoethnographic account of transforming from a dismissed patient to a ‘knower’ through writing, revealing how ECT‐induced amnesia and compulsory care undermined personal narratives.
- The paper challenges psychiatry's neutrality, showing how values like rationality tend to diminish experiential wisdom and highlights recovery as restoring agency beyond standardized assessments.

**What are the implications for mental health nursing?:**

- Questions that invite patients to explain their own experiences could foster shared inquiry, replacing judgement with curiosity and power‐sharing.
- Recognizing adaptive responses as valid knowledge can rebuild trust, integrate patient perspectives into care and support recovery.

## 1 How This Account Is Written

This paper is presented as an autoethnography, written as a reflexive personal narrative. It draws on personal memory, diary notes, journal entries from several inpatient admissions and later reflective writing developed through revisiting those notes. These materials capture ward experiences as they unfolded and how they were reinterpreted over time.

Analytically, I situate the account within literature on epistemic injustice (Fricker 2007) and lived‐experience epistemologies. Fricker's concept of testimonial injustice helps explain how labels such as ‘lack of insight’ can diminish the credibility of experiential testimony, while mad knowledge literature (Beresford 2025; Voronka 2017) frames coping behaviours as interpretive practices rather than as symptoms alone. Terms such as ‘impaired reflective capacity’ may likewise operate as credibility‐diminishing mechanisms in clinical interactions.

Although this account takes a critical view of diagnostic practice, it does not suggest that psychiatry is monolithically oppressive. Rather, it highlights recurring institutional patterns while recognizing variation in clinician intentions, local policy constraints and resource limitations. The critique is therefore structural rather than personal. In the following section, these patterns take on a more immediate form. What appears at first as an abstract institutional critique takes shape in one particular situation, at one particular table, where language, gaze and clinical framing shape the encounter before I can present my own understanding of it.

## 2 The Ward Encounter

The psychiatric ward room carried the sharp scent of bleach mixed with stale instant coffee. Two doctors sat opposite me at a wooden table, their notebooks separating us physically and symbolically. Before I even spoke, I was measured and categorized by their eyes, their faces settling into that familiar mix of pity and frustration. In this room, I was told things. Things about who I was. About what I felt. ‘You're in denial’ the oldest one said while his pen scratched steadily across the page. To them, my anorexia was proof of collapse, a textbook sign of distorted thinking and even psychosis. To me, it was something else entirely—an armour against flashbacks that struck like fists. I clung to control through hunger when memories threatened to tear my world apart. But instead of searching for meaning, they dissolved my experience into a list of symptoms. What I experienced as survival became pathology, boxed into the DSM like groceries checked off a list. There was an obvious chasm between my understanding and theirs—no common ground, only contrasting interpretations. That sterile moment distilled everything inpatient life would keep repeating: My account was undervalued because it came from the patient's side of the table, an instance of testimonial injustice (Fricker 2007) in which my credibility was reduced not by the content of what I said, but by my position within the psychiatric system. From a mad knowledge perspective (Beresford 2025; Voronka 2017), which foregrounds the credibility of lived experience often dismissed in clinical settings, starvation instead emerges as an embodied interpretive practice—a way of managing overwhelming trauma rather than an inert expression of illness.

During my years in inpatient care, labels piled on. Self‐harming, dissociative, aggressive, manipulative, attention‐seeking. I swallowed their biomedical language whole before I could question it, undoubtedly internalizing a sick identity. The credibility crisis (cf. Fricker 2007) came during my 15th hospitalization in 2007. It peaked with 47 ECT treatments crammed into 4 months. Electrodes clamped to temples. Currents surged. Light exploded and vanished again. I woke into a suffocating mental blur with severe retrograde amnesia. I could not name the white walls boxing me in. I could not recall relationships, conversations and whole years of my life. My story had been surgically erased, yet my body still remembered terror without context. It became clear that the doctors saw the obliteration of memory as proof of illness rather than as an adverse effect of treatment.^1^ They documented ‘impaired reflective capacity’, marking my cognitive fog as a sign of depression instead of consequence. They spoke of chronicity, not iatrogenic harm (cf. Elwyn 2023; Moberg 2022). I still remember the doctor's phrase: ‘You don't seem to have the ability to tell your own story’. Shame pierced the haze, and I wondered whether I was truly *that* broken. Was my lost memory proof of a defective mind? That verdict trailed me into compulsory care for starvation—the same script replayed again. Irrational denial is a classic marker of ‘mental illness’. Yet, I had starved consciously, trying to reclaim control when all other threads had snapped.

Then, one day—why, I cannot say—a stray thought cut through the fog: *I will make something of this*. Sitting on the ward, suspended in that half‐detached existence, I sensed, however faintly, that the suffering itself might contain the seed of understanding. That I, too, was a knower.

## 3 Breaking Silence Through Writing

From that enforced passivity rose a quiet, defiant frustration, an inner shift that would much later find a form through writing. That moment became the hinge between despair and inquiry—the flicker of autoethnography (cf. Peterson 2017; Summers 2021) before I recognized it as such. My hard‐won ways of managing pain were recast as dubious flaws, defects to be fixed rather than stories to be understood. The cognitive scars followed me into social work classes where textbooks blurred into hieroglyphs through paralyzed concentration. I scraped through anyway, sheer stubbornness pushing me past what logic insisted was impossible. A doctoral programme followed and my cognition still scarred yet determined. I wanted to prove that comprehension could rise from rupture.

The turning point came in October 2022. My laptop's blue light spread across scattered notes—yellow pads covered with ward memories, journal fragments and ECT aftermath recorded in frantic handwriting. I had lived within the routines and surveillance of inpatient care, where time dissolved into medication rounds and observation schedules. Now, as a doctoral researcher, I had been given the space to reshape those experiences into an autoethnographic work. I began to type the first scene, tracing the texture of that life from the inside out. Each sentence prompted recognition and helped integrate fragmented memories. Personal knowledge, once dismissed as illness logic, re‐emerged as embodied coping responses, evidence tempered through ordeal. I was now a witness, speaking back.

After that act of writing, my knowledge began to deepen. The older wound demanded something different—a slow unlearning of the obedience that the institution itself had bred. I had to question every label, peel back the patient role and confront how ‘care’ had shaped my own self‐image. It meant stripping diagnostic language down to its bones and realizing that what psychiatry had called a shortcoming was often a logic of endurance—an intelligence that had gone unrecognized. By the time I stood in a lecture room as a doctoral student, that endurance had become fuel. I carried the ward's echo into every class, not as trauma but as curriculum. I began teaching recovery as a collision—lived wisdom meeting clinical knowledge head‐on, both reshaping the other. Patient voices, still resonating from locked wards, offered maps of care that psychiatry still failed to read, dismissing them as diagnostic noise. That ward room no longer haunted me as defeat but as buried truth uncovered. My knowledge had always made sense within my own reality; it had only collided violently with the biomedical gaze and its narrow expectations of what ‘mentally ill’ people could know or sound like.

## 4 Towards a Curious Practice

Looking back, I see that no knowledge system is ever neutral. At times, psychiatry cloaks itself in scientific objectivity, yet its foundations rest on unspoken values: rationality as virtue, control as proof of health and normality as the gatekeeper of credibility (cf. Fricker 2007). Those ideals consolidate power behind invisible walls. When other kinds of knowing—experiential, embodied, emotional—press against those walls, cracks begin to spread. True recovery can be understood as reclaiming agency where passivity was enforced, restoring equality where power was seized and rebuilding trust in one's own interpretation after years of having it dismantled. These are not abstract ideals but concrete acts of reconstruction.

One question could have sparked it all: ‘How do you understand your experience?’ That simple pivot—from expert verdict to shared inquiry—might have reframed that entire trajectory from my first admission day in September of 2004, when partnership could have meant judgement was replaced by curiosity and compliance by mutual learning. For mental health professionals asking how this shift from judgement to curiosity might look in practice, I offer three concrete examples from what I needed but did not receive. In *initial assessment conversations*, rather than beginning with standardized symptom checklists, the encounter could start with an invitation to describe how the person understands what is happening. When I sat across from those two doctors with my anorexia reduced to ‘denial’ and ‘distorted thinking’, no one asked what starvation protected me from or why I could not live without it. Had a professional responded with curiosity—‘What does that control shield you against?’—My survival strategy might have been recognized as embodied wisdom rather than me being viewed as unknowing or irrational.

Further, *in documentation practices*, professionals can include patients' own explanations alongside clinical observations. When patients' perspectives disappear from the official record, future staff inherit only the pathologizing narrative, not the person behind it. Finally, *in care planning*, goals should be co‐designed rather than imposed. What would have felt helpful to me was someone asking ‘What would feel like progress for you?’ Rather than becoming a constructive starting point, the situation instead developed into recurring conflicts between staff and me, often culminating in coercive measures such as forced feeding and restraint. These practices require time and institutional support, but small shifts are possible even within resource limitations. In its absence, however, my ‘recovery’ was flattened into numbers: BMI curves tracked like stock prices, assessment scores tallied into neat graphs that missed the point entirely. Those graphs reflected a belief in what counted as knowledge and reality.

On that day in 2022, I began questioning that reality. When engaging in autoethnography, I transformed private persistence into shared testimony, bringing survival and subjectivity into a broader interpretation (cf. Fox 2021). Where medicine had erased a story, writing rebuilt it as proof of life. The act itself was reclamation. Later, I came to see recovery differently: It was not primarily about compliance or having others define it for me but about regaining a sense of agency and continuity. My understanding of myself and my experience shifted substantially, marking an important change in how I related to knowledge and identity. Care can become strained when patients speak from lived experience in ways that challenge professional assumptions. This was truly my experience. Through writing, I came to recover not only from distress but also from the reducing impact of psychiatric practice (cf. Punzi and Bergstrand 2024). This suggests a double recovery: first, moving beyond the position of passivity, and second, beginning to recover from the system that had reinforced that position. Today, some of my students ask where change within psychiatry can begin. I believe the answer is straightforward. It begins with curiosity, with listening, with the willingness to hear what survival tried to accomplish, no matter how unfamiliar or unfiltered it sounds. Progress can be traced not only in assessment scales but also in small recognitions—moments when mutual understanding takes root. When power is shared and dialogue matters more than verdict, care begins to transform. In those pale corridors, I always knew something that psychiatry could not measure. My insight diverged from diagnostic frameworks but carried truth all the same. The path to improvement lies there, in giving that kind of knowing a seat at the table. Mad knowledge (cf. Beresford 2025; Voronka 2017) does not patch over medicine. Rather, it reshapes its foundations, suggesting that healing requires recognition and participation. Putting these experiences into words became a crossing—a way to move between clinical language and lived reality. It helped me evolve from being defined by others to becoming a knower, from object to author. This turning point echoes through every lecture I give: Patients should not be reduced to diagnostic deficits. Rather, they are interpreters of experience, charting terrain professionals have not always learned to map. The telling itself has become a form of healing that others can join.

Writing this autoethnographic account has been part of my process of creating space to reinterpret experiences that were previously dismissed or framed by professionals. More importantly, it suggests that survival can constitute a form of epistemic resource when lived experience is taken seriously. Recognizing this may support more collaborative and person‐centred practices in psychiatric care.

## Ethics Statement

This study draws upon a lived‐experience narrative. As it does not involve research participants, ethics approval was not required.

## Conflicts of Interest

The author declares no conflicts of interest.

## Data Availability

Data sharing not applicable to this article as no datasets were generated or analysed during the current study.

## Endnotes

^1^
Reflections on electroconvulsive treatment in this paper are presented as my lived experience and interpretation, not as a general evaluation of ECT's efficacy or indications.
